# Speciation Analysis of Arsenic Compounds by High-Performance Liquid Chromatography in Combination with Inductively Coupled Plasma Dynamic Reaction Cell Quadrupole Mass Spectrometry: Application for Vietnamese Rice Samples

**DOI:** 10.1155/2019/5924942

**Published:** 2019-04-28

**Authors:** Hai Anh Vu, Manh Ha Nguyen, Hong-An Vu-Thi, Quan Do-Hong, Xuan Hoang Dang, Thi Ngoc Bich Nguyen, Hong Quan Trinh, Thuy Ly Bich, Tien-Thanh Nguyen, Dung Le-Van, Minh Binh Tu, Dinh Binh Chu

**Affiliations:** ^1^Faculty of Chemistry, VNU University of Science, Vietnam National University Hanoi, 19 Le Thanh Tong, Hoan Kiem, Hanoi 100000, Vietnam; ^2^Department of Analytical Chemistry, School of Chemical Engineering, Hanoi University of Science and Technology, 1 Dai Co Viet, Hai Ba Trung, Hanoi 100000, Vietnam; ^3^Institute of Chemistry, Vietnam Academy of Science and Technology (VAST), 18 Hoang Quoc Viet, Cau Giay, Hanoi 100000, Vietnam; ^4^School of Environmental Science and Technology, Hanoi University of Science and Technology, 1 Dai Co Viet, Hai Ba Trung, Hanoi 100000, Vietnam; ^5^School of Biotechnology and Food Technology, Hanoi University of Science and Technology, 1 Dai Co Viet, Hai Ba Trung, Hanoi 100000, Vietnam; ^6^Department of Chemistry, Vietnam Military Medical University, 160 Phung Hung, Ha Dong, Hanoi 100000, Vietnam

## Abstract

In this work, high-performance liquid chromatography in combination with inductively coupled plasma dynamic reaction cell quadrupole mass spectrometry was introduced and optimized for speciation analysis of five major arsenic species including arsenobetain (AsB), arsenite (As(III)), monomethylarsonic (MMA), dimethylarsenonic acid (DMA), and arsenate (As(V)) in rice samples. Five arsenic compounds were separated on a Hamilton PRP X100 strong anion-exchange column employed with the mobile phase that is compatible with mass spectrometry, containing ammonium carbonate, methanol, and disodium ethylenediaminetetraacetic acid. Arsenic compounds were detected online by inductively coupled plasma dynamic reaction cell quadrupole mass spectrometry utilizing oxygen as the reaction gas at a flow rate of 0.7 mL·min^−1^. Five selected arsenic species were baseline separated at the optimum experimental conditions. The excellent LOD and LOQ values of the developed method were achieved in the range of 0.5 to 2.9 *μ*g·kg^−1^ and 1.7 to 9.6 *μ*g·kg^−1^ for all species of arsenic, respectively. The ionization effect in plasma during chromatographic gradient elution was systematically investigated by using postcolumn injector. Arsenic compounds in rice samples were extracted by diluted nitric acid at elevated temperature. The extraction efficiency and the interconversion of target compounds during sample preparation were also assessed. The full validation of the developed method was performed by using certified reference material, BRC 211, from European Institute of Reference and Standard for speciation analysis. The recovery of all selected arsenic species was in the range of 70 to 135.5%. The validated method was also applied to analyze rice samples collected from some contaminated rice fields. The results showed that As(III), DMA, and As(V) were found in all rice samples. Average concentration (range) of inorganic arsenic and DMA in all rice samples were 130.3 (65.5–228.1) and 32 (8.2–133.01) *μ*g·kg^−1^, respectively. However, total concentration of inorganic arsenic in most of investigated rice samples was below the maximum residual level according to US-FDA and European Union standards.

## 1. Introduction

Rice, especially white rice, is the most staple food in the diet of Vietnamese people. It is an indispensable ingredient of traditional meals in Vietnam as well as in some other Asian countries. In Vietnam, rice is the most common crop grown on agriculture land with total production of approximate 29 million tons in 2018, accounted for around 6% of the total rice production in the world, which puts Vietnam in the fourth position amongst the top rice producing countries, after China, India, and Bangladesh. [1] However, in some agricultural areas, rice is grown in flooded soil or water contaminated with arsenic compounds. Therefore, obtaining information about total arsenic concentration, especially concentration of arsenic species, is particularly important in assessing potential exposure risk of arsenic and its species to human health. The European Union and United State of American Food and Drug Administration approved a tolerance level of inorganic arsenic (iAs) as 0.2 mg·kg^−1^ and 0.1 mg·kg^−1^ for polished rice and rice-based products for infants and young children, respectively. The human exposure to arsenic compounds via rice consumption has been also investigated [2, 3]. In Vietnam, unfortunately, the reported data of arsenic in rice samples was very limited, especially for the concentration of arsenic species [4–6]. Therefore, it is necessary to have a selective, sensitivity, reliable, and validated analytical method for both total arsenic contents and its species in Vietnam rice samples.

Recently, many analytical methods have been introduced for quantification of arsenic species in the rice sample such as gas chromatography tandem mass spectrometry after appropriate derivatization steps, and liquid chromatography in combination with atomic absorption/fluorescence spectrometry (HPLC-AAS/AFS) [7, 8], liquid chromatography in combination with atomic absorption spectrometry via hydride generator (HPLC-HG-AAS), liquid chromatography in combination with inductively coupled plasma quadrupole mass spectrometry (HPLC-ICP-QMS) [6, 9, 10], gas chromatography mass spectrometry, and gas chromatography atomic emission spectrometry have been also introduced for speciation analysis of arsenic compounds in ready-to-eat rice samples [11, 12]. Moreover, a nonchromatographic separation was also introduced for analysis of inorganic arsenic in rice samples [13]. Among these methods, HPLC-ICP-QMS is the most popular for speciation analysis of arsenic compounds in both environmental and biological samples because of its selectivity, sensitivity, and flexibility [14–16].

In this work, speciation analysis of arsenic compounds was conducted on Vietnamese rice samples collected from rice fields in Red River Delta, especially in some contaminated soil areas. Arsenic species in rice samples were extracted with appropriate solvents and separated by anion-exchange chromatography employed with gradient elution. Arsenic compounds were identified by both retention time on the anion-exchange column and arsenic monoxide tracer (AsO^+^*m*/*z* 91) in ICP-DRC-QMS. In order to overcome isobaric mass interferences in ICP-DRC-QMS, dynamic reaction cell employed with oxygen gas was used. Ionization suppression or enhancement in HPLC-ICP-DRC-QMS for speciation of arsenic compounds was assessed via a postcolumn flow injector. All parameters of both anion exchange chromatography and inductively coupled plasma quadrupole mass spectrometry were optimized in order to achieve the highest selectivity and sensitivity. Fully validation of both total and speciation analysis of arsenic compounds was performed by using commercially available certified reference materials. Finally, the developed method was applied to speciation analysis of arsenic compounds in Vietnamese rice samples that were collected from some rice fields in Red River Delta of Vietnam.

## 2. Materials and Methods

### 2.1. Materials

Arsenobetaine (AsB, purity ≥ 95.0%), cacodylic acid (dimethylarsinic acid: DMA, purity ≥ 98%), and disodium methyl arsonate hexahydrate (monomethylarsonic acid, MMA, purity ≥  98%) were purchased form Sigma Aldrich (Singapore). Arsenite stock standard (As(III), 1000 mg/L as As) in 2% HCl and arsenate stock standard (As(V), 1000 mg/L as As) in H_2_O were collected from PerkinElmer (USA). Nitric acid (≥65%), ammonium carbonate (99.999% trace metals basis), ammonium hydroxide solution (28% NH_3_, purity ≥ 99.99% trace metals basis), disodium ethylenediaminetetraacetic acid (ACS reagent, purity 99.4–100.6%, powder), methanol for HPLC (gradient grade, suitable as ACS-grade LC reagent, purity ≥ 99.9%), and ethylenediaminetetraacetic acid disodium salt (EDTA, analytical grade) were purchased from Sigma Aldrich (Singapore). Deionized water (DIW), 18 MΩ·cm, from Millipore Milli-Q system, was used for preparation of standard solutions, samples, and mobile phase solutions. Single arsenic stock solutions were prepared in DIW and kept at −20°C in polypropylene tubes. Working solutions were fresh daily prepared by dilution of stock solutions in DIW. Certified Reference Material (CRM): arsenic in rice flour (ERM-BC211) from European Reference Materials, Institute for Reference Materials and Measurements (Geel, Belgium), which is certified for the concentration of inorganic arsenic and DMA, as well as total arsenic, was used for validation of the developed analytical method for speciation analysis of arsenic compounds in rice samples.

Mobile phase solution on channel A was prepared as follows: add 0.24 g (NH_4_)_2_CO_3_ and 0.025 g EDTA to a 1 L plastic HPLC reservoir containing 900 g DIW; next, adjust the pH to 9.00 (±0.05) with 28% ammonium hydroxide and then add 50 mL of methanol and finally fill to 1000 g with DIW. For channel B, 2.4 g (NH_4_)_2_CO_3_ and 0.025 g EDTA were added to a 1-liter plastic HPLC reservoir containing 990 g DIW, and the remaining steps were similar to the preparation of solution on channel A. Prior to use, all mobile phase solutions were filtered through a 0.45 *μ*m polyvinyldifluoride membrane filter and degassed by using ultrasonic bath. Mobile phase solutions were prepared fresh daily in order to minimize changes in pH caused by effects from the environment.

### 2.2. Instruments

Separation of arsenic compounds was carried by an Altus™ A-30 UPLC® System (PerkinElmer, USA) including an Altus A-30 solvent delivery module (metal-free quaternary pump) and Altus A-30 sample injection (an autosampler equipped with a 100 *μ*L injection loop and silica-coated needle). It also has an online degasser and a column thermostat compartment. The HPLC system is controlled by Empower 3 version 7.0.3471.909 from Water Corporation (Water, MA, US). Arsenic compounds were separated on PRP-X100 strong anion-exchange column (4.6 × 150 mm, 5 *μ*m particle size; Hamilton, NV, USA) employed with gradient elution. Arsenic compounds were detected by NexION 2000 ICP-DRC-QMS (Perkin Elmer, USA) as a specific elemental detector. For detection, arsenic compounds eluting out of the strong anion-exchange column were directly sent to the ICP-DRC-QMS system via polyether ether ketone (PEEK) liquid chromatography tubing. The arsenic species were detected by arsenic monoxide (^75^As^16^O^+^, *m*/*z* 91) in dynamic reaction cell mode utilizing oxygen as the reaction gas. The identification of arsenic species was performed by comparing the retention times of single standards on RPR Hamilton X100 at the given chromatographic conditions and the tracer of ^75^As^16^O^+^ on the ICP-DRC-QMS. The sensitivity and selectivity of the ICP-DRC-QMS were daily checked by using specific tuning solution from PerkinElmer (N9303843, PerkinElmer, USA). Sensitivity of arsenic measurement was optimized and checked daily by using 10 ng·mL^−1^ As(V) standard solution (Merck, Singapore). The operating conditions of both HPLC and ICP-DRC-QMS are showed in Table 1.

### 2.3. Collection and Preparation of Rice Samples for Speciation Analysis

Rice samples were collected mainly from several provinces in Vietnam such as Hanam, Haiphong, and Nghe An. Some other samples were randomly collected in a supermarket in Hanoi. Total 23 samples were collected and transported to the laboratory for further pretreatment. Rice samples were rinsed with double distilled water to remove dust and then dried by air flow at room temperature. These samples were then freeze-dried at −50°C in the FreeZone 4.5 Liter benchtop freeze dryers (Labconco, MO, USA). Next, rice was ground into powder by using a commercially available blender with stainless steel blades. Finally, the powdered rice samples were kept in sealed plastic bags at 4°C until analysis.

### 2.4. Sample Preparation

#### 2.4.1. Total Analysis of Arsenic

0.2 g of freeze-dried rice samples was weighed into a 75 mL Teflon microwave digestion vessel, and 6 mL of concentrated nitric acid was added. The vessel then was allowed to stand overnight. Afterward, the rice sample was digested in a microwave oven (Multiwave PRO 50 HZ Package 24HVT8, Anton PAAR, Graz, Austria). The temperature program was increased up to 165°C in 15 min and then held at this temperature for a further 10 min. In the second step of the digestion, the vessels were cooled to 50°C for 20 min. After digestion, the samples were cooled to room temperature, transferred quantitatively into 50 mL volumetric flasks, and made up to volume with DIW. These solutions were then analyzed for the total arsenic content by ICP-DRC-QMS. Rice-based certified reference material (ERM BC-211) and fish-based certified reference materials (DORM 2, DORM 4, and BRC 627) were used for quality control and validation of the analytical method for total arsenic analysis.

#### 2.4.2. Speciation Analysis

Rice samples preparation followed a procedure that was proposed by US-FDA with some modifications. In brief, 1.0 g of the ground rice was transferred into a 50 mL centrifuge tube. And then, 5 mL of 0.28 M HNO_3_ was added, followed by quick vortex mixing and rinsing the walls of the container with the another 5 mL of 0.28 M HNO_3_. Next, all tubes were capped tightly and placed in a preheated block digestion system at 95°C for 90 min. After that, samples were let to cool and diluted by adding deionized water. Samples were then centrifuged at 1.3 × 10^4^ g for 10 min, and supernatants were filtered using a 0.45 *μ*m nylon syringe filter. These solutions were subjected for analysis via HPLC-ICP-DRC-QMS at the optimum operating conditions as follows. For quality control, rice-based matrix certificated reference material (ERM-BC 211 rice flour, European Union Breaux of Standard; Brussels) was prepared in the same manner as described above and analyzed at the same time. The extraction efficiency was calculated by comparing results between measurements and certified values. Practically, the number of quality control and blank samples accounted for 20% of the total sample subjected for analysis in both total and speciation analysis. The triplicates of sample and blank also included in the batch of analysis. For monitoring the carry-over effect, blank sample that contained mobile phase was injected after every ten injections into HPLC-ICP-DRC-QMS.

## 3. Results and Discussions

### 3.1. Total Arsenic in Rice Samples

Rice samples were digested in a microwave oven and analyzed for total concentration arsenic by ICP-DRC-MS using indium as an internal standard. In order to validate ICP-MS-based analytical method for quantification of total arsenic in real samples, three fish-based matrix and one rice-based matrix certified reference materials were used. 0.2 g of certificated reference materials (DORM 2 and DORM 4 from CRM Canada, ERM-BRC 627 from European Union) and rice-based certificated reference material (ERM-BC 211 from European Union) were acid-digested in a microwave oven and analyzed by using ICP-DRC-QMS. The concentration of total arsenic in DORM 2, DORM 4, BRC-627, and ERM BC 211 was 17.3 ± 0.3 mg·kg^−1^, 7.13 ± 0.1 mg·kg^−1^, 4.56 ± 0.05 mg·kg^−1^, and 255 ± 12 *μ*g·kg^−1^, respectively. The certified total concentrations of arsenic are 18 ± 1.1 mg·kg^−1^, 6.84 ± 0.44 mg·kg^−1^, 4.8 ± 0.3 mg·kg^−1^, and 260 ± 13 *μ*g·kg^−1^ for DORM 2, DORM 4, BRC-627, and ERM BC 211, respectively. The experimental results indicated that no significant difference was observed between measured and certified values in this study. It should be noted that the ICP-DRC-QMS-based analytical method in combination with microwave-assisted acidic digestion was the method that had an intended purpose for analysis of total arsenic in rice. Total concentration of arsenic in rice samples are listed in Table 2. As can be seen from this table, the concentration of arsenic in rice ranged from 124.6 ± 9.8 *μ*g·kg^−1^ to 407 ± 39.6 *μ*g·kg^−1^.

### 3.2. High-Performance Liquid Chromatography for Separation of Arsenic Compounds

For speciation analysis of arsenic compounds, in this study, anion-exchange chromatography was directly combined with ICP-DRC-QMS via 60 mm PEEK tubing. Arsenic compounds were separated on an anion-exchange column (Hamilton PRP X100, 150 × 4.6 mm × 5 *μ*m) by employing with gradient of mobile phase compatible with mass spectrometry, including ammonium carbonate, EDTA, and methanol as organic modifiers. Arsenic compounds were detected via an arsenic monoxide tracer (^75^As^16^O^+^) by ICP-DRC-QMS employed with oxygen as the reaction gas. The separation of five arsenic species in this work was modified from our previous studies [17, 18]. In brief, five arsenic species were separated on the strong anion-exchange column with gradient of mobile phase, which was described as follows: the concentration of methanol and EDTA in the mobile phase was 5% and 0.05%, respectively. The following mobile phase program is the optimum gradient for separation of five arsenic species. The concentration of ammonium carbonate (pH 9.0) was kept at 5 mM for first one minute and linearly increased to 50 mM (pH 9.0) in the next 11 minutes. Then, it was kept at 50 mM for 4 minutes before being decreased to 5 mM in 0.1 minutes. Finally, the concentration of 5 mM was kept for 6 minutes in order to regenerate the column for the next injection. Total time for chromatographic separation was 22 minutes. The flow rate of mobile phase was maintained constantly at 1 mL·min^−1^. Due to the fact that no structure information of arsenic compounds in ICP-DRC-QMS was obtained, identification of arsenic compounds was assessed via retention time on the Hamilton PRP X100 column and the data of arsenic monoxide tracer in ICP-DRC-QMS when analyzing single compounds under optimized conditions as described above.

**Figure 1:**
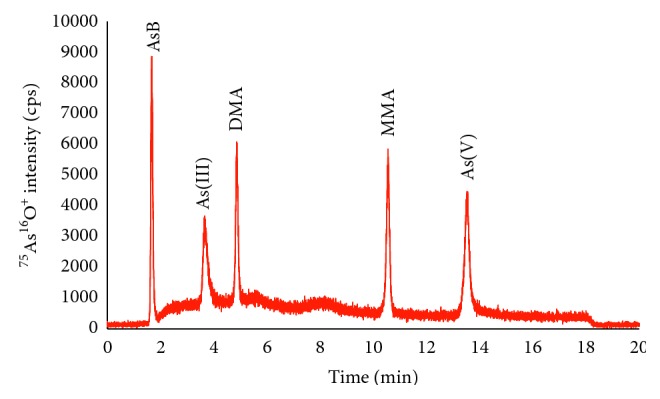
Chromatogram of five arsenic species separated on the Hamilton PRP-X100 strong anion-exchange column. Concentration of all species: 1 ng·mL^−1^. Other experimental conditions are listed in Table 1.

As is illustrated in the chromatograph in Figure 1, five arsenic species were baseline separated under the optimum chromatographic conditions. The observed order of elution was AsB, As(III), DMA, MMA, and As(V) on the Hamilton PRP X-100 column. The resolution of all species ranged from 2.47 to 10.16. It was worth noting that the chromatographic resolution of As(III) and DMA pair was 2.47. This information was so important because both As(III) and DMA were present in some rice samples as described in Table 2. Other critical parameters of the chromatographic separation including symmetry and peak width at baseline were also calculated according to European Pharmacopeia (Section 2.2.46 Chromatographic separation techniques) and listed in Table 3.

#### 3.2.1. Column Recovery

For assessment of column recovery, the standard solutions containing five arsenic species as well as five single standard solutions were injected into the HPLC-ICP-DRC-QMS system at the optimum experimental conditions. The same volume of standard solutions was injected into the ICP-DRC-QMS system at the same experimental operating conditions via a flow injector employed with 5 mM of ammonium carbonate containing 5% methanol as carrier. The column recovery of all species was calculated by dividing total peak area in LC-DRC-QMS by area of the peak obtained after flow injection into ICP-DRC-QMS. As can be seen from the results in Table 3, column recovery of five arsenic species was ranged from 85.3 to 98.9%. It could be concluded that the elution strength of the selected mobile phase was strong enough to elute all species out of the analytical column. In addition, the selected mobile phase components showed the good separation and detection of all selected arsenic species in this study.

### 3.3. Optimization of ICP-DRC-QMS for Detection of Arsenic

Many analytical methods have been developed and introduced for quantitation of total arsenic content in environmental and biological samples. Among them, inductively coupled plasma quadrupole mass spectrometry is one of the most popular methods for total arsenic analysis because of its selectivity, sensitivity, multielement analysis, and isotope dilution analysis. However, the presence of interferences such as isobaric mass interferences or polyatomic mass interferences in ICP-QMS is common issue, which led them to be one of the critical parameters, especially in low-resolution mass spectrometry like quadrupole mass spectrometer, affected on the robustness of the analytical method. Many polyatomic ions, e.g., ^40^Ar^35^Cl^+^, ^59^Co^16^O^+^, ^36^Ar^38^Ar^1^H^+^, ^38^Ar^37^Cl^+^, ^36^Ar^39^K^+^, ^43^Ca^16^O_2_^+^, ^23^Na^12^C^40^Ar^+^, ^12^C^31^P^16^O_2_^+^, are overlapped on the low-resolution mass spectrum of arsenic [19]. In this study, the PerkinElmer Nexion 2000 ICP-DRC-QMS system is equipped with three modes: standard/collision cell/dynamic reaction cell (DRC) was used for arsenic measurement. In DRC mode, oxygen gas was used as the reaction gas, and ^75^As^16^O^+^ (*m*/*z* 91) was monitored instead of the As^+^ (*m*/*z* 75) tracer. The most important parameter in DRC mode is reaction gas flow (or reaction gas pressure in dynamic reaction cell). For the assessment of the influence of gas flow on both total and speciation analyses, the oxygen flow was changed from 0.5 to 0.9 mL·min^−1^ to 0.1 mL·min^−1^ step. The optimum flow of oxygen was obtained at 0.7 mL·min^−1^ for both sensitivity and repeatability. Therefore, flow of oxygen gas was kept at 0.7 mL·min^−1^ for further experiments.

#### 3.3.1. Ionization Effect in Inductively Coupled Plasma

**Figure 2:**
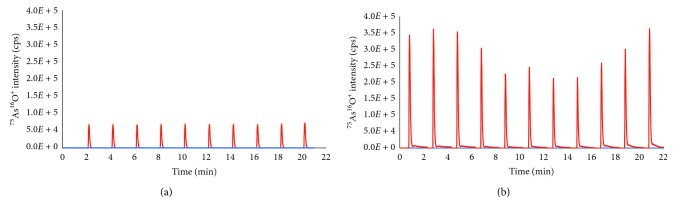
Flow injection transient signals of arsenic intensity performed via a species-unspecific postcolumn injector. The blue and red lines present blank and transient signals of the analyte in both figures. (a) Transient signal of arsenic (As(V)) in deionized water as the carrier; (b) transient signal of arsenic (As(V)) in gradient of mobile phase as the carrier.

For investigation of the ionization enhancement and suppression in plasma employed with high carbon content mobile phase, a 100 ng·mL^−1^ solution of arsenate in 2% nitric acid (Merck, Singapore) was used. The postcolumn injection was performed manually via a postcolumn injector every two minutes. The postcolumn injector and instrumental setup were more detailed in the previous work [20]. The species-unspecific solution was injected every two minutes, using the eluent containing only deionized water or gradient of mobile phase. The transient signals of these experiments in water and mobile phase are depicted in Figures 2(a) and 2(b), respectively.

It is clear from Figure 2(a) that the signal of the analyte was kept constant for the whole time of chromatographic separation process if deionized water was used as carrier. Meanwhile, in Figure 2(b), the analytical signal was strongly dependent on the gradient of the mobile phase (gradient of concentration of ammonium carbonate in the mobile phase) during chromatographic separation time. When the concentration of ammonium carbonate in mobile phase was low, the analytical intensity of arsenic was high. In contrast, when higher concentration of ammonium carbonate in mobile phase was applied, the lower analytical intensity was observed. It could be attributed to the increase of carbon in plasma that can change the physiochemical properties of plasma (Figure 2(b)). In addition, the analytical signal of the target analyte (peak area and peak height) in the mobile phase was enhanced at least 2 to 4 times higher than that in deionized water. It could be explained by the enhancement of analytical signal in the presence of carbon in the plasma source, which led to a phenomenon known as charge transfer reaction between the target analyte and C^+^ or ArC^+^. This effect occurred in the plasma source as proposed by Kovačevič et al. [21–23].

#### 3.3.2. Analytical Figure of Merits of the HPLC-ICP-DRC-QMS for Speciation Analysis of Arsenic Compounds

Table 3 lists the chromatographic characteristics of the optimized method, such as retention time, chromatographic resolution peak symmetric, and peak width at baseline according to European Pharmacopeia. Other critical parameters of the developed method are repeatability of the retention time and analytical signal (peak area). For the assessment of short-term stability of the retention time and peak area, three independent solutions of five arsenic species were freshly prepared in deionized water and injected into the HPLC-ICP-DRC-QMS system. For long-term stability, one mixture of five arsenic species was prepared and continuously measured for 20 hours in HPLC-ICP-DRC-QMS. Relative standard deviation of retention time and peak area of all arsenic species was calculated and listed in Table 4. The stability of retention time was achieved in the range of 0.2–3.0% (short term, *n*=3) and 0.15–6.76% (long-term stability) for all AsB, As(III), DMA, MMA, and As(V). The experimental results demonstrated that the excellent repeatability of retention time was achieved for separation of arsenic species via anion-exchange chromatography. However, the retention time shift was observed after approximately 100 injections of samples (especially real samples). Therefore, in this case, the column was regenerated by back flush overnight using a solvent as per the suggestion of the manufacturer. The repeatability of analytical signal is a parameter that has a significant contribution on the uncertainty of measurements. The repeatability of peak areas of all arsenic species was assessed by injecting an arsenic standard mixture solution at different times. Relative standard deviations of peak areas of five arsenic compounds were in the range of 2–16% and 4–17% for short-term and long-term stability, respectively. It was worth noting that the good repairability of peak areas was achieved in this study.

Other parameters of the analytical method are limit of detection and limit of quantitation. For the assessment of LOD and LOQ of the introduced method, six standard solutions with concentration of all arsenic species from 0.5 to 10 ng·mL^−1^ were prepared and injected in triplicate into HPLC-ICP-DRC-MS systems. The calibration functions were calculated by linear regression, based on average peak areas and concentration of the target analyte. LOD and LOQ were calculated as the concentration at the signal–to-noise ratio of 3 and 10, respectively, according to US-FDA guidance and other works [24–29].

As can be seen from Table 5, the excellent correlation between analytical signal and concentration of arsenic species was achieved. LOD and LOQ of the developed method were high enough for directly analysis of arsenic species in rice samples. Absolute LOD and LOQ of the developed method were achieved at picogram levels. LOD and LOQ of the developed method were also comparable with recent publications [9, 30]. In summary, the introduced and optimized method was fit-for-purpose regarding the quantification of arsenic compounds in rice samples.

#### 3.3.3. Interconversion during Sample Preparation

For investigation of conversion during the sample preparation, spiking experiments were carried out by adding standard solutions into rice samples before extraction. Five single standard solutions of each arsenic compounds at the concentration of 20 ng·mL^−1^ was used for spiking experiments. The results have shown that only conversion of As(III) to As(V) was observed (approximate 30% of As(III) changed to As(V) in such experimental conditions). The conversion of As(III) to As(V) was also confirmed in the work of Narukawa et al. [31]. However, it could not be affected on the total concentration of inorganic arsenic in rice samples because it was calculated by the sum of concentration of As(III) to As(V).

### 3.4. Validation of the Developed Method

**Figure 3:**
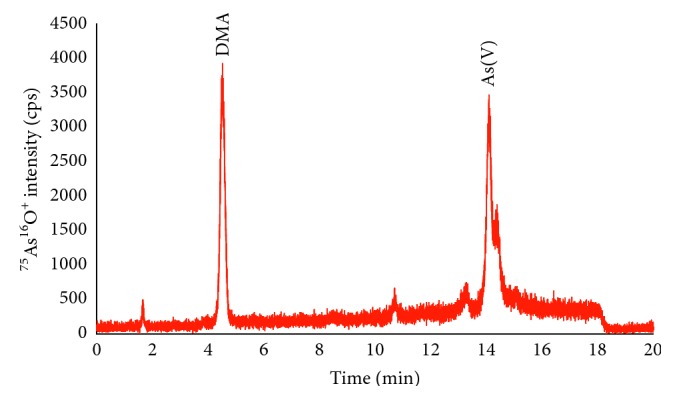
HPLC-ICP-DRC-QMS chromatogram of arsenic species in certified reference materials ERM-BC 211.

The full validation of the developed method is performed by either comparison with another validated method or using certified reference material according to Guidance from United Stated Food and Drug Administration. In this study, the validation was carried out by using rice-based ERM-BC211 certified reference material from European Reference Materials. The ERM-BC211 was prepared and analyzed at the optimum conditions for both approaches: total and speciation analysis. The HPLC-ICP-DRC-QMS chromatogram of the ERM-BC211 is depicted in Figure 3, and the concentration of total arsenic and arsenic species is shown in Table 6.

As clearly shown in Table 6, experimental results and certified values of both total and species concentration of arsenic indicated the good agreement between them in this work. It is concluded that the introduced method was fit-for-purpose for quantitation of total and speciation analysis of arsenic compounds in rice. In addition, the extraction efficiency of other arsenic compounds like AsB and MMA that do not present in the certified reference material was calculated by spiking experiments. Rice samples were spiked with 20 ng·g^−1^ standard mixture solution of five arsenic species and treated in the same manner as described above. Recovery of AsB, As(III), DMA, MMA, and As(V) was 105%, 70%, 108%, 98.4%, and 135.5%, respectively. The lower recovery of arsenite in rice samples was explained by interconversion during sample preparation as mentioned above and proposed in the published work [32]. Nevertheless, total inorganic arsenic content in rice samples was calculated by summing up concentration of arsenite and arsenate. Therefore, interconversion of arsenite to arsenate did not affect the quantitation of total inorganic arsenic content in rice samples.

### 3.5. Analysis of Arsenic Compounds in Rice Samples

**Figure 4:**
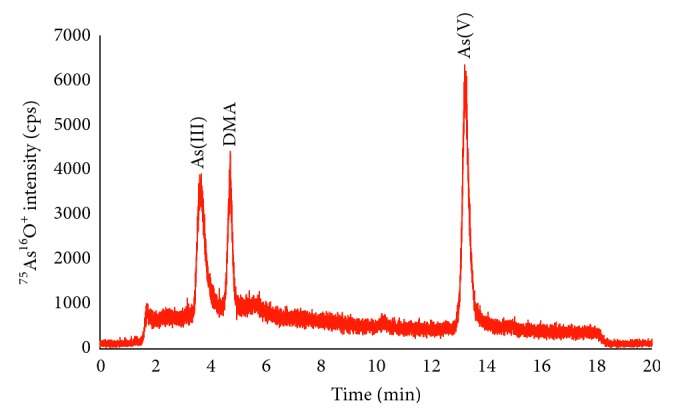
HPLC-ICP-DRC-QMS chromatogram of arsenic species in rice samples.

For the application of the proposed method, total 23 rice samples were collected and analyzed as the aforementioned protocol. The representative chromatogram of arsenic compounds in rice samples is indicated in Figure 4.

The concentration of arsenic species as well as total arsenic determined by ICP-DRC-MS after acidic digestion in the microwave oven in rice samples are demonstrated in Table 6. Total inorganic arsenic was calculated by the sum of As(III) and As(V).

As can be seen from Table 2, three arsenic species, As(III), DMA, and As(V), were detected in all rice samples. AsB or unretained species like tetramethylarsonium and MMA were found at low concentration in only few samples. The presence of tetramethylarsonium in rice samples which contained elevated arsenic concentration was also found by Hansen et al. [33] or it can be the product of interaction between arsenic species with mobile phase [34]. Percentage of inorganic arsenic compounds was calculated by dividing total inorganic arsenic by total arsenic content analyzed by ICP-DRC-MS after acidic digestion in the microwave oven. The percentage of inorganic arsenic (iAs) and DMA in all rice samples ranged from 46.9 to 94.1% and from 7.7 to 36.7%, respectively. The mean values and corresponding standard deviation of As(III), DMA, As(V), and iAs in all analyzed rice samples were 83.3 ± 28.1, 32 ± 26.7, 46.9 ± 35.5, and 130.2 ± 67.8 *μ*g·kg^−1^, respectively. Concentration of iAs was found higher than 200 *μ*g·kg^−1^ in only one rice sample (R18), and it could be explained that this sample was taken from rice field nearby mining activity area. However, concentration of iAs in rice samples was most below maximum residual level according to US-FDA and European Union standards [35, 36].

## 4. Conclusion

In this work, a high-performance liquid chromatography in combination with inductively coupled plasma dynamic reaction cell mass spectrometry has been successfully introduced and optimized for speciation analysis of arsenic compounds in rice samples. The analytical signal of arsenic during chromatographic separation was also investigated via a postcolumn injector. The experimental results demonstrated that the analytical signal of arsenic strongly depended on the gradient of mobile phase. The developed method has been fully validated by rice-based certificated reference material. This validated analytical method was used for analysis of arsenic species in 23 rice samples collected from rice fields in Red River Delta, Vietnam. Two inorganic arsenic species including arsenite and arsenate were found in some rice samples. However, total inorganic arsenic in the most samples was below US-FDA and EU standards. In the next study, assessment of human exposure to arsenic species through rice consumption and other sources, e.g., water and fish-based food, will be presented.

## Figures and Tables

**Table 1:** Operating conditions of HPLC-ICP-DRC-QMS for speciation of arsenic compounds.

Operating conditions of HPLC-ICP-DRC-QMS	
*High-performance liquid chromatography operating conditions*	
Pump	Altus A-30 quaternary pump equipped with solenoid solvent selection valve, online degasser, liquid autosampler with 100 *µ*L injection loop, and column temperature compartment
Column	PRP X 100, 4.6 × 150 mm, 5 *μ*m particle size; Hamilton, NV, USA
Column temperature	Room temperature (app. 25°C)
Eluent	Channel A: 5 mM (NH_4_)_2_CO_3_, pH 9.0, 0, 05% EDTA, 5% MeOH
	Channel B: 50 mM (NH_4_)_2_CO_3_, pH 9.0, 0, 05% EDTA, 5% MeOH
Elution mode	Gradient elution: 0-1 minute: 100% A and then increasing linearly to 100% B in 10 minutes and kept for 4 minutes. Finally decreasing to 100% in 0.1 minutes and kept for 6 minutes to regenerate column
Flow rate	1.0 mL·min^−1^
Injection volume	50 *μ*L
*ICP-DRC-QMS operating conditions*	
RF power	1600 W
Plasma gas flow	15 L·min^−1^
Auxiliary gas flow	1.2 L·min^−1^
Nebulizer gas	1 L·min^−1^
Nebulizer	PFA
Spray chamber	Quartz cyclonic
Sample cone	Platinum sample cone
Skimmer cone	Platinum hyper skimmer cone
Cell entrance voltage	−7 V
Cell exit voltage	−7 V
DRC mode	Dynamic reaction cell with oxygen gas, 0.7 mL·min^−1^
Monitored ion	AsO^+^ (*m*/*z* 91)
Measurement mode	Peak hopping
Dwell time	100 ms
RPq	0.6
Quantification mode	External calibration curve

**Table 2:** Concentration (*μ*g·kg^−1^ dried weight, mean value ± SD, *n*=3) of arsenic species and total arsenic in rice samples.

No.	Sample ID	AsB or unretained species	As(III)	DMA	MMA	As(V)	Total iAs^*∗*^	Total^*∗∗*^	Total^*∗∗∗*^
1	R1	41.04 ± 1.22	132.23 ± 4.27	133.01 ± 4.80	2.71 ± 0.08	53.62 ± 5.59	185.85 ± 7.03	362.84 ± 17.91	407.24 ± 39.56
2	R2	106.26 ± 3.17	101.35 ± 3.27	63.71 ± 2.30	2.99 ± 0.09	51.55 ± 5.38	152.9 ± 6.3	325.89 ± 11.94	353.24 ± 19.75
3	R3	30.29 ± 0.9	100.24 ± 3.23	38.47 ± 1.39	<LOD	1.13 ± 0.12	101.37 ± 3.23	170.62 ± 5.3	154.42 ± 12.04
4	R4	18.62 ± 0.56	99.36 ± 3.21	22.57 ± 0.81	2.83 ± 0.09	0.59 ± 0.06	99.95 ± 3.21	143.25 ± 4.64	140.11 ± 10.93
5	R5	15.9 ± 0.47	104.14 ± 3.36	59.44 ± 2.14	<LOD	0.43 ± 0.05	104.57 ± 3.36	180.26 ± 5.95	179.73 ± 14.02
6	R6	12.77 ± 0.38	8.97 ± 0.29	30.86 ± 1.11	<LOD	105.2 ± 10.97	114.17 ± 11	157.03 ± 12.19	148.5 ± 11.58
7	R7	<LOD	99.02 ± 3.19	36.39 ± 1.31	<LOD	38.9 ± 4.06	137.92 ± 5.2	174.31 ± 8.38	189.5 ± 14.78
8	R8	<LOD	12.03 ± 0.39	30.52 ± 1.10	<LOD	100.6 ± 10.49	112.63 ± 10.5	143.14 ± 11.77	153.94 ± 12.01
9	R9	<LOD	79.47 ± 2.56	14.73 ± 0.53	<LOD	39.43 ± 4.11	118.9 ± 4.84	133.63 ± 6.95	142.68 ± 11.13
10	R10	<LOD	111.63 ± 3.6	17.68 ± 0.64	<LOD	65.73 ± 6.86	177.36 ± 7.75	195.04 ± 10.84	236.01 ± 18.41
11	R11	<LOD	93.94 ± 3.03	33.05 ± 1.19	<LOD	34.55 ± 3.6	128.49 ± 4.71	161.53 ± 7.56	169.83 ± 13.25
12	R12	<LOD	75.8 ± 2.45	8.71 ± 0.31	<LOD	63.39 ± 6.61	139.19 ± 7.05	147.91 ± 9.18	156.36 ± 12.2
13	R13	<LOD	94.63 ± 3.05	13.90 ± 0.50	<LOD	71.68 ± 7.48	166.31 ± 8.1	180.21 ± 10.82	187.56 ± 14.63
14	R14	<LOD	86.52 ± 2.79	46.66 ± 1.68	<LOD	71.03 ± 7.41	157.55 ± 7.9	204.21 ± 11.68	211.93 ± 16.53
15	R15	<LOD	84.35 ± 2.72	20.01 ± 0.72	<LOD	42.78 ± 4.46	127.13 ± 5.2	147.31 ± 7.73	151.02 ± 11.78
16	R16	<LOD	68.55 ± 2.21	26.16 ± 0.94	<LOD	52.14 ± 5.44	120.69 ± 5.9	146.85 ± 8.38	154.36 ± 12.04
17	R17	<LOD	93.5 ± 3.02	25.82 ± 0.93	<LOD	64.99 ± 6.78	158.49 ± 7.4	184.31 ± 10.52	191.87 ± 14.97
18	R18	<LOD	98.7 ± 3.18	41.72 ± 1.50	<LOD	129.31 ± 13.49	228.01 ± 13.9	269.73 ± 18.02	283.11 ± 22.08
19	R19	<LOD	84.55 ± 12.07	21.02 ± 2.37	<LOD	29.07 ± 0.76	113.62 ± 12.1	134.64 ± 12.3	283.90 ± 22.23
20	R20	<LOD	86.69 ± 12.38	10.88 ± 1.23	<LOD	43.53 ± 1.14	130.22 ± 12.4	141.1 ± 12.5	176.50 ± 13.82
21	R21	<LOD	57.19 ± 8.16	8.22 ± 0.93	<LOD	16.70 ± 0.44	73.89 ± 8.2	82.11 ± 8.2	124.60 ± 9.76
22	R22	<LOD	64.51 ± 9.21	21.27 ± 2.40	<LOD	0.98 ± 0.03	65.49 ± 9.2	86.76 ± 9.5	152.20 ± 11.92
23	R23	<LOD	78.20 ± 11.16	10.92 ± 1.23	<LOD	1.02 ± 0.03	79.22 ± 11.2	90.14 ± 11.2	150.70 ± 11.80

^*∗*^Total inorganic arsenic was calculated by sum of arsenite and arsenate; ^*∗∗*^total arsenic was calculated by sum up five species; ^*∗∗∗*^total arsenic was determined by acidic digestion in the microwave oven and measured by total analysis.

**Table 3:** Characteristics of the chromatographic separation of arsenic species on the Hamilton PRP X100 column.

No.	Analyte	Retention time (min, mean value ± SD, *n*=3)	Resolution (mean value ± SD, *n*=3)	Symmetry (mean value ± SD, *n*=3)	*W* (min, mean value ± SD, *n*=3)	Column recovery (%, value ± SD, *n*=3)
1	AsB	2.00 ± 0.15	—	1.51 ± 0.14	0.410 ± 0.033	85.3 ± 1.0
2	As(III)	3.54 ± 0.25	2.97 ± 0.11	2.01 ± 0.27	0.560 ± 0.019	95.6 ± 0.5
3	DMA	5.10 ± 0.10	2.47 ± 0.35	1.29 ± 0.18	0.488 ± 0.052	86.7 ± 1.5
4	MMA	10.53 ± 0.01	10.16 ± 0.26	0.86 ± 0.02	0.623 ± 0.040	97.9 ± 2.1
5	As(V)	14.06 ± 0.03	5.25 ± 0.24	0.99 ± 0.10	0.842 ± 0.032	98.9 ± 0.5

Resolution =  1.18 *∗* (*t*_R2_ − *t*_R1_)/*w*_h1_+*w*_h2_; symmetry = *w*_0.05_/2 *∗* *d* according to European Pharmacopeia.

**Table 4:** Short-term and long-term stability of retention times and peak areas.

No.	Analyte	Retention time			Peak area	
		Mean value (min), *n*=3	RSD of short-term (%), *n*=3	RSD of long-term (%),	RSD of short-term (%), *n*=3	RSD of long-term (%)
1	AsB	2.00	1.28	6.76	15.92	16.66
2	As(III)	3.54	3.02	3.36	14.28	14.57
3	DMA	5.10	1.99	2.80	11.29	12.65
4	MMA	10.53	0.13	0.15	2.86	4.19
5	As(V)	14.06	0.19	0.40	2.63	8.03

**Table 5:** Analytical figure of merits of speciation analysis of five arsenic species in HPLC-ICP-DRC-QMS.

No.	Analyte	Regression equation	*R* ^2^	LOD (ng·g^−1^) (mean value ± SD, *n*=3)	LOQ (ng·g^−1^) (mean value ± SD, *n*=3)	LOD^a^ (pg) (mean value ± SD, *n*=3)	LOQ^a^ (pg) (mean value ± SD, *n*=3)
1	AsB	*y* = (83200 ± 1300) ∗ *x* − (63400 ± 56500)	0.9988	0.5 ± 0.1	1.7 ± 0.3	25.8 ± 4.2	85.9 ± 13.9
2	As(III)	*y* = (48900 ± 600) ∗ *x* − (35100 ± 28200)	0.9991	2.9 ± 0.3	9.6 ± 0.9	144.2 ± 46.2	480.6 ± 154
3	DMA	*y* = (52900 ± 700) ∗ *x* − (44200 ± 32300)	0.9990	0.7 ± 0.2	2.4 ± 0.6	36.0 ± 8.5	119.8 ± 28.5
4	MMA	*y* = (49000 ± 700) ∗ *x* + (41600 ± 15700)	0.9992	0.9 ± 0.1	3.1 ± 0.4	46.1 ± 5.6	153.8 ± 18.6
5	As(V)	*y* = (56100 ± 300) ∗ *x* + (9600 ± 13600)	0.9998	0.9 ± 0.2	2.9 ± 0.6	42.8 ± 9.0	146.0 ± 30

^a^Absolute amount of the analyte injected into the column.

**Table 6:** Concentration of arsenic species and total concentration in certified reference material (ERM-BC 211).

No.	Analyte	Concentration (*μ*g·kg^−1^)	
		Experimental results (mean value ± SD, *n*=5)	Certified valued (certified value ± U, *k* = 2)
1	DMA	123.45 ± 4.45	119 ± 13
2	As(III)	<LOD	nc^a^
3	As(V)	134.79 ± 10.93	nc^a^
4	Total iAs	134.79 ± 10.93	124 ± 11
5	Total As	258.95 ± 16.05	260 ± 13

^a^Concentration of As(III) and As(V) is not shown in the certification of analysis of ERM-BC 211.

## Data Availability

The LC-ICP-DRC-MS chromatograms and all data used to support the findings of this study are available from the corresponding author upon request.
